# Trace Elements in Hermann’s Tortoises (*Testudo hermanni*) According to Sex, Season, and Sampling Region in Central Europe

**DOI:** 10.3390/ani14152178

**Published:** 2024-07-26

**Authors:** Christoph Leineweber, Gregor Geisler, Michael Pees, Sabine Öfner, Rachel E. Marschang

**Affiliations:** 1 Laboklin GmbH & Co. KG, Steubenstrasse 4, 97688 Bad Kissingen, Germany; 2 Department of Small Mammal, Reptile and Avian Medicine and Surgery, University of Veterinary Medicine Hannover, Bünteweg 9, 30559 Hanover, Germany; 3 Reptile Rescue Center Munich e.V., Kaulbachstrasse 37, 80539 Munich, Germany

**Keywords:** trace elements, tortoise, reference interval, sex, season, region

## Abstract

**Simple Summary:**

Some trace elements are essential for the health of vertebrates, but only limited data are available on blood levels in tortoises. The aim of this study was therefore to measure trace elements in heparinized blood plasma of Hermann’s tortoises (*Testudo hermanni*) (*n* = 520) from March to September 2022 using inductively coupled plasma mass spectrometry (ICP-MS) and to establish specific reference intervals. Additionally, the influence of sex, season, and region of sample collection on the measured values were evaluated. Significant (*p* ≤ 0.05) sex-specific, seasonal, and regional differences were found for a number of trace elements indicating that all of these factors should be considered when establishing and interpreting blood trace element levels in tortoises.

**Abstract:**

Some trace elements are essential for the health of vertebrates, but little is known about their function, the amounts required, and the factors influencing their metabolism in tortoises. The aim of this study was therefore to measure trace elements (chromium (Cr), cobalt (Co), copper (Cu), iron (Fe), magnesium (Mg), manganese (Mn), molybdenum (Mo), selenium (Se), zinc (Zn)) in heparinized blood plasma of Hermann’s tortoises (*Testudo hermanni*) (*n* = 520) from March to September 2022 using inductively coupled plasma mass spectrometry (ICP-MS) and to establish specific reference intervals. Additionally, the influence of sex, season, and region of sample collection on the measured values were evaluated. Significant (*p* ≤ 0.05) sex-specific differences were found for Cu, Mg, and Mn; seasonal differences were found for Cr, Cu, Mn, Mo, and Se; and the region in which the tortoises were kept significantly impacted Cr, Cu, Fe, Mg, Mo, and Se levels. The results show that all of these factors should be consider when establishing and interpreting blood trace element levels in tortoises.

## 1. Introduction

Wild populations of Hermann’s tortoises (*Testudo hermanni* [GMELIN, 1789]) occur in littoral pinewoods, coastal dunes, mediterranean scrub, and garrigues in Mediterranean Europe [1]. The western subspecies *Testudo hermanni hermanni* is found in eastern Spain, southern France, the Balearic Islands, Corsica, Sardinia, and central Italy, while the eastern subspecies *Testudo hermanni boettgeri* is more common in Croatia, North Macedonia, Romania, Bulgaria, Albania, Greece, and the European part of Turkey [1]. Their natural diet consists of legumes (e.g., clover and lupins) and diverse plants of the families *Poaceae*, *Rubiaceae*, *Scrophulariaceae*, *Cruciferae*, *Ranunculaceae*, *Araliaceae*, *Asteraceae*, and *Rosaceae*, among many others [2]. In contrast, Hermann’s tortoises under human care in Germany are generally kept in combined outdoor and greenhouse enclosures and fed combinations of local wild herbs (e.g., dandelion, red clover, and plantain weed), lettuce, hay, and rarely other vegetables, fruit, and small invertebrates [3]. A study in Hermann’s tortoises showed that the dietary calcium (Ca) content influences intestinal resorption of both Ca and magnesium (Mg) [4].

Little is known about blood trace elements in reptiles, especially trace elements like chromium (Cr), cobalt (Co), copper (Cu), iron (Fe), Mg, manganese (Mn), molybdenum (Mo), selenium (Se), and zinc (Zn), which have been shown to be essential for the metabolism and health in mammals [5,6,7]. In livestock, Co is needed for the synthesis of vitamin B12, which in turn is an important component in the metabolism of carbohydrates, lipids, and amino acids, as well as in the synthesis of DNA [5,6]. Fe is an important component in hemoglobin and myoglobin and is therefore of particular importance for oxygen transport in the body [5,6]. Cu plays an important role as a structural component and part of enzymes and is therefore important for the immune system, growth, and the nervous system. As an enzyme component, Zn influences cell division and thus growth as well as immune function [5,6]. Mo is important as an enzyme component in the metabolism but also as an antagonist of Cu [5,6]. Se is contained in a number of enzymes including glutathione peroxidase (GPX) and plays an important role as an antioxidant in this context [5,6]. Mn is also an important enzyme component involved in carbohydrate, lipid, and protein metabolism [5,6]. All of these trace elements must be ingested with the diet, and especially in herbivores, deficiencies or intoxications occur due to strongly fluctuating proportions in the diet. It is therefore important to monitor whether the animals are adequately supplied as well as to monitor possible oversupply. The easiest way to do this is to analyze the elements in the animal’s blood. However, many elements are also stored in the body, so that amounts of these trace elements in the blood may reflect only the currently circulating concentrations [5,6].

In Hermann’s tortoises, multiple studies have documented blood levels of the macroelements Ca and phosphate (P) [8,9,10,11,12,13,14,15], and one study included Mg levels [10]. There is, however, very little information on the levels of microelements in the blood of any tortoise species or on the role of these elements in the health of reptiles. There is a single study that examined Cu, Fe, Zn, Se, and Mn levels in the blood of Hermann’s tortoises [16]. In this study, serum samples from 130 clinically healthy Hermann’s tortoises from different locations (reptile rescue center in southern Germany, extensively kept tortoises from Turkey and free ranging tortoises from two locations in France) were analyzed using different laboratory techniques (spectrophotometry for Cu, Fe, and Zn and atomic absorption spectrometry for Se and Mn) [16].

Sex and season have been shown to strongly influence the blood levels of the macroelements Ca, P, and Mg in Hermann’s tortoises [9,10,13,15]. A single study on microelements in Hermann’s tortoises also documented sex-specific variations in the blood levels of Cu, Mn, and Zn as well as seasonal influences for Fe, Mn, and Se [16]. Seasonal influences are due not only to hormonal changes depending on the season but also due to differences in the available food spectrum depending on the season as well as the hibernation of the animals in winter, during which time no food is consumed, and the metabolism is slowed down. The region in which animals are kept may also influence the mineral content in the food due to differences in mineral content of the soil. A study in Hermann’s tortoises [16] was able to document differences depending on the region in which the animals were kept for Cu, Se, and Zn. In this study, the lowest Zn levels were measured in Germany and the highest in Turkey, whereas Cu and Se were lowest in Turkey and highest in France (Cu), respectively Germany (Se) [16].

The aim of this study was therefore to establish reference intervals for a wide range of trace elements in the heparinized plasma of Hermann’s tortoises under human care in Central Europe. Reference intervals are important to enable the correct interpretation of blood results and provide a basis for further studies to research the clinical relevance of trace elements in tortoises. We hypothesized that sex, season, and the region in which the animals live would have a significant influence on the trace element levels found in the plasma. 

## 2. Materials and Methods

### 2.1. Animals

**Figure 1:**
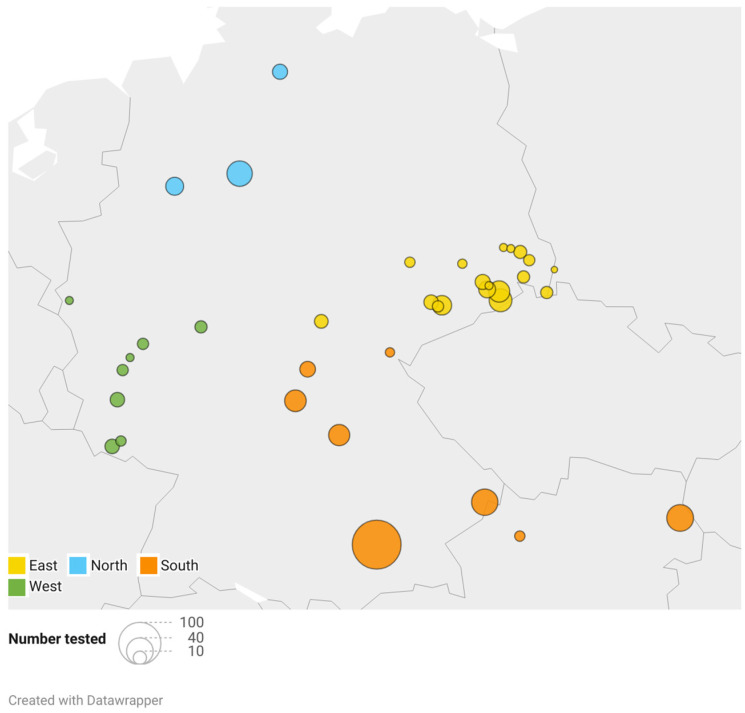
Geographical distribution and number of samples analyzed. The color coding shows the classification of the locations to the respective region, and the size of the dots correlates with the number of animals sampled.

Blood samples from clinically healthy adult Hermann’s tortoises (318 males, 202 females) were collected during clinical health checks from March to September 2022. The sampling was approved by the ethics commission of the Faculty of Veterinary Medicine of the University of Leipzig (GZ: EK 21/2021). The body weight of the Hermann’s tortoises ranged from 110 g to 7000 g (mean ± SD = 1014 ± 578 g). Blood samples were grouped according to the season in which they were collected: March to May was considered spring (*n* = 93), June to July early summer (*n* = 210), and August to September late summer (*n* = 217). This categorization corresponds to the standard definitions of each season, without reference to specific temperature in the year of sampling. It reflects changes in temperature and length of days and the corresponding changing dietary spectrum and activity of the animals. Due to the hibernation of the tortoises, no samples were collected in fall or winter. The tortoises were kept in naturalistic environments as pets by private keepers as well as in public zoological institutions for exhibition and also included animals that were given up or found and kept in reptile rescue centers. Animal collections sampled were distributed over 35 different locations all over Germany and 2 locations in Lower Austria (Figure 1). The locations were grouped in north (3 locations; total samples *n* = 58), west (8; *n* = 42), east (18; *n* = 149), and south (8; *n* = 271), according to their geographical distribution (Figure 1). Tortoises were fed ad libitum with wild herbs collected in the surroundings, hay, and hay cobs, as well as small portions of leafy greens, vegetables, and fruits, some of which originated from other regions. Some of the animals were kept in greenhouses paned with simple window glass, while others were kept outdoors with direct access to unfiltered sunlight. 

All tortoises were considered healthy on the basis of a general health check and showed no changes on the shell, skin, or nails and no softening or abnormal formations of the carapace at the time of blood collection. Animals sampled in spring had all begun eating following hibernation and all were considered in good body condition. 

### 2.2. Sample Collection and Analysis

The blood samples were collected mostly from the dorsal coccygeal vein, in some cases from the subcarapacial plexus with 3 mL syringes with a Luer system (Omnifix^®^ Luer Lock Solo, B. Braun SE, elsungen, Germany) and canulae (Sterican^®^ blue 23 G × 3 1/8””, Ø 0.6 × 30 mm, B. Braun SE, Melsungen, Germany). No samples with visible lymph contamination or a PCV < 10% were included in the study. A total of 0.5 to 3.0 mL blood was collected depending on the animal’s body weight and never exceeded 0.7% of the total body weight. The blood was then transferred into lithium-heparinized tubes (4.5 mL tube, lithium heparin, Sarstedt AG and Co. KG, Nurnbrecht, Germany) and transported in an upright position overnight cool (4–8 °C, 39.2–46.4 °F) to the laboratory. Samples were centrifuged at 3220× *g* for 3 min in a Thermo Scientific Megafuge ST Plus Series (Thermo Fisher Scientific Inc., Breda, The Netherlands) no later than 24 h after collection. The subsequent examinations requiring heparin plasma were performed within 24 h after centrifugation, and plasma samples were stored cool (8 °C; 46.4 °F) until testing. The trace elements Cr, Co, Cu, Fe, Mg, Mn, Mo, Se, and Zn were measured in triplicate using a 1:51 dilution with 1% nitric acid (HNO3) solution using inductively coupled plasma mass spectrometry (ICP-MS) (ICPMS-2030, Shimadzu Germany GmbH, Duisburg, Germany) [17]. Before the plasma was analyzed, the instrument was calibrated using the ClinCal serum Calibrator (RECIPE Chemicals + Instruments GmbH, Munich, Germany), and germanium and terbium in specific concentrations were added to each sample as internal standards [17]. The limit of detection (LOD) and the limit of quantification (LOQ) for each element were as follows: Cr (LOD < 0.03 µg/L; LOQ < 0.1 µg/L), Co (LOD < 0.06 µg/L; LOQ < 0.2 µg/L), Cu (LOD < 0.003 mg/L; LOQ < 0.01 mg/L), Fe (LOD < 0.03 mg/L; LOQ < 0.1 mg/L), Mg (LOD < 0.03 mg/L; LOQ < 0.1 mg/L), Mn (LOD < 0.3 µg/L; LOQ < 1.0 µg/L), Mo (LOD < 0.3 µg/L; LOQ < 1.0 µg/L), Se (LOD < 0.3 µg/L; LOQ < 1.0 µg/L), and Zn (LOD < 0.06 mg/L; LOQ < 0.2 mg/L). The calculated mean value of the triplicate measurement for each element from each animal was used for the statistical analyses. Measured levels that were below the lower quantification limit of the test were set at half of the quantification limit for the statistical analysis. 

### 2.3. Statistical Analyses

Statistical analyses were calculated using the SAS and SPSS analysis software package (SAS OnDemand for Academics; SAS Institute Inc., Cary, NC, USA and SPSS 28.0; IBM, Armonk, NY, USA). A Shapiro–Wilk test was used for the determination of normal distribution. A linear model with a univariate variance analysis was used to evaluate the influence of the factors sex, season, and region on the measured trace elements and the interactions between these factors. The cut off for significant for these models was set at *p* < 0.05 with Bonferroni correction and an α of 0.05 (Supplementary Material). Reference intervals (RIs) were determined according to the guidelines of the American Society of Veterinary Clinical Pathologists (ASVCP) [18] using Reference Value Advisor v2.1 and the nonparametric method [19]. Outliers were determined using Tukey and Dixon–Reed tests.

## 3. Results

The calculated reference intervals for each element for all Hermann’s tortoises are shown in Table 1. The variance analysis showed that the sex of the tortoise, the season in which the blood sample was collected and the region in which the tortoises were kept significantly influenced the plasma levels of several of the elements measured. Therefore, specific reference intervals for each sex, each season, and each region were calculated if significant differences were found (Table 1).

**Figure 2:**
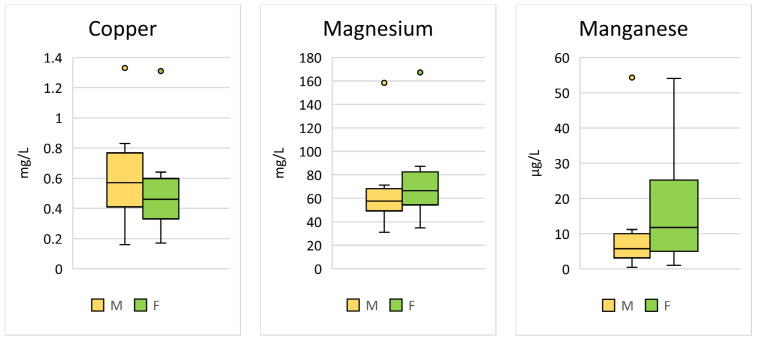
Comparison of the trace elements in Hermann’s tortoises (*Testudo hermanni*) that were significantly (*p* < 0.05) influenced by the sex of the tortoise (male (M) and female (F)). Box-plots show the median, 10th and 90th percentiles, minimum, and maximum.

Significant sex differences were found for Cu (*p* < 0.001; F = 24.45), Mg (*p* < 0.001; F = 30.02), and Mn (*p* < 0.001; F = 48.75) (Table 2, Figure 2).

**Figure 3:**
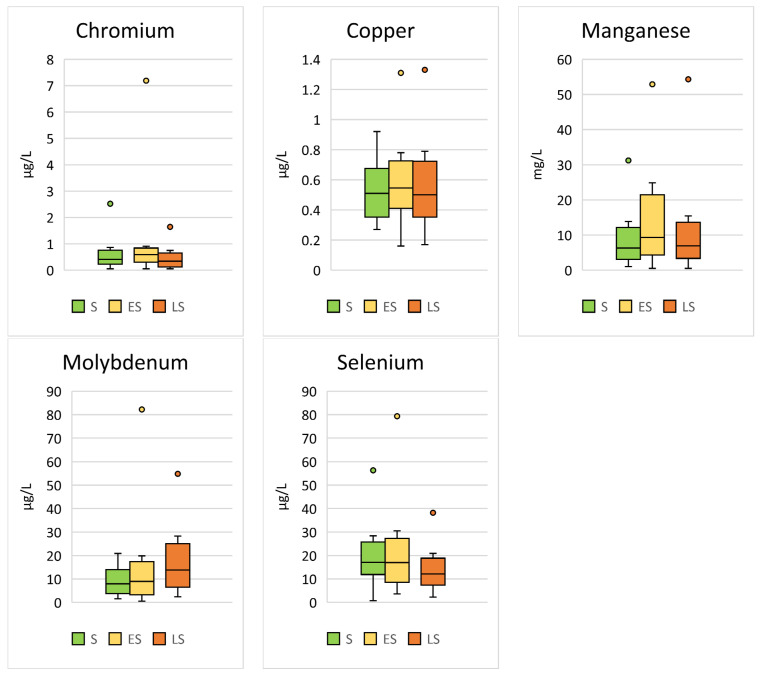
Comparison of the trace elements in Hermann’s tortoises (*Testudo hermanni*) that were significantly (*p* < 0.05) influenced by the season in which the blood sample was collected (spring (S), early summer (ES), and late summer (LS)). Box-plots show the median, 10th and 90th percentiles, minimum, and maximum.

Significant seasonal differences were found for Cr (*p* < 0.001; F = 13.18), Cu (*p* < 0.001; F = 7.05), Mn (*p* < 0.001; F = 7.66), Mo (*p* < 0.001; F = 21.64), and Se (*p* < 0.001; F = 21.20) (Table 3, Figure 3).

**Figure 4:**
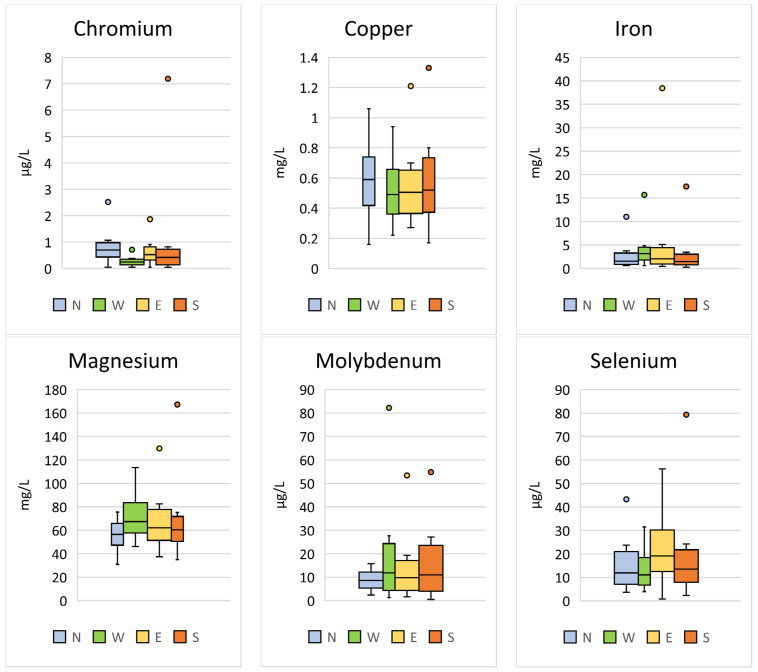
Comparison of the trace elements in Hermann’s tortoises (*Testudo hermanni*) that were significantly (*p* < 0.05) influenced by the region in which the tortoises were kept (north (N), west (W), east (E), and south (S)). Box-plots show the median, 10th and 90th percentiles, minimum, and maximum.

Significant differences between the region of sample collection were found for Cr (*p* < 0.001; F = 17.88), Cu (*p* = 0.003; F = 4.73), Fe (*p* = 0.040; F = 2.80), Mg (*p* < 0.001; F = 7.04), Mo (*p* < 0.001; F = 9.31), and Se (*p* < 0.001; F = 9.87) (Table 4, Figure 4).

Significant interaction between the factors were found for the combinations of sex × season for Cr (*p* = 0.018; F = 4.06) and Mn (*p* = 0.005; F = 5.31); for the combination of sex × region for Se (*p* < 0.001; F = 8.25); for the combination of season × region for Cr (*p* < 0.001; F = 9.21), Mg (*p* = 0.004; F = 5.46), Se (*p* < 0.001; F = 25.04), and Zn (*p* < 0.001; F = 14.16); and for the combination of sex × season × region for Mg (*p* = 0.039; F = 3.27) and Zn (*p* = 0.019; F = 4.02).

Significant positive correlations with the weight of the turtles were found for Cu (*p* < 0.001; 0.17), Mn (*p* < 0.001; 0.37), and Se (*p* < 0.001; 0.25), while negative correlations with weight were found for Co (*p* = 0.002; −0.12) and Zn (*p* < 0.001; −0.20).

## 4. Discussion

Trace elements have multiple functions for the metabolism and the health of animals. They play a role as components and cofactors of enzymes, proteins, and vitamins [5,6,7]. A deficiency can therefore trigger various clinical signs including anemia; bone abnormalities; skin problems; weakened immune system; and gastrointestinal, muscular, and neurological disorders [5,6,7]. On the other hand, many are only required in relatively small amounts, and high intakes can lead to intoxication [5,6,7]. However, little is known about physiological levels of various trace elements in the blood of tortoises. Evaluation of trace element requirements and physiological blood levels is further complicated by the effects of hormonal changes depending on the sex and the season as well as variations in the food spectrum and the intake of trace elements depending on the season and the region in which an animal lives [16]. These factors can influence the levels in the blood of the tortoises. In the present study, it could be shown that not all elements are equally influenced by all factors.

In comparison to a previous study that also measured some trace elements in Hermann’s tortoise [16], the measured median levels for Fe (0.44 mg/L) and Zn (1.70 mg/L) were lower, and the levels for Cu (0.50 mg/L), Mn (6.85 µg/L), and Se (15.95 µg/L) were similar in the present study. In a study on spur-tight tortoises *(Testudo graeca*) in Libya [20], lower mean levels for Mg (males 56.4 mg/L; females 53.3 mg/L) were measured in heparinized plasma in comparison to the values measured in the present study. Some of the tortoises from a German rescue center were tested in a previous study [16] as well as in the present study in different years, but in the previous study [16], serum and different measurement methods were used, and both studies also included tortoises from other locations. These differences in location are most likely associated with differences in mineral content in the ground and vegetation as well as in the diets of the tortoises, which could explain the differences in calculated ranges between the studies. The study from Libya [20] also used a different detection method for Mg. The advantage of the ICP-MS method used in the present study is that only a small amount of sample material (100 µL) is required, and a large number of elements can be measured simultaneously [21]. Furthermore, the method is very accurate and is less influenced by hemolysis or lipemia than other methods such as spectrophotometry [21]. In comparison to the Mg levels measured from the same samples using spectrophotometry (median 58.3 mg/L; range 29.2–77.8 mg/L) [10], the levels measured by ICP-MS (median 60.0 mg/L; range 31.0–167.2 mg/L) were significantly (*p* < 0.001) higher. Caution should therefore be used when comparing the results of different measurement methods with each other, as well as between different sample materials as some elements are higher in whole blood than in plasma and there can also be different concentrations between serum and plasma depending on the element [22]. 

Sex-specific variations in trace element levels were found for Cu, Mg, and Mn in the present study. A previous study [16] also found that the sex of Hermann’s tortoises influenced serum levels of Cu, Mn, and Zn, whereby the values for Zn were not influenced by sex in the present study. A study on spur-thighed tortoises in Turkey found higher Fe values in males, especially in the pre-reproduction period in comparison to females, and an increase in Fe in females during the reproduction period [23]. No sex-specific differences were found for Mg in spur-thighed tortoises [18,21]. The sex-specific differences in the elements have various causes, but the main cause is likely the hormonally influenced differences in metabolism, absorption, mobilization, and excretion of elements, especially during the reproductive phase [16,23,24]. The influence of the weight of animals on the measured trace elements is also related to the sex, as adult females are larger than males of the same age, but the age plays a crucial role [25]. The tortoises in the study were adult, but the age of many tortoises is not exactly known due to changes in ownership and differences in the growth rates of individual tortoises depending on the environmental conditions.

Trace elements also varied seasonally in the blood of the tortoises. Cr, Cu, Mn, Mo, and Se all showed significant differences between the seasons in the present study. A previous study on Hermann’s tortoises also found seasonal fluctuations for Fe, Mn, and Se [16]. In this study [16], the documented increases in Se levels from spring to summer followed by decreases to fall were similar to the results of the present study. Mn increased in the present study from spring to early summer but decreased in the previous study [16] from spring to fall. The seasonal differences are partly hormonal, as female tortoises, for example, need and mobilize minerals for egg production [24,26], but also because the supply and the composition of food plants changes throughout the year [1,2,3]. Some plants may also contain anti-nutritional substances like tannins, oxalates, and phytate, which reduce the bioavailability and the resorption of some trace elements and can cause deficiencies [27].

The region or habitat in which the tortoises live influence the trace element levels, because the mineral content of the soil in which a plant grows affects the plant mineral content. This and the variation of different food plants in the different regions and habitats influences the mineral intake of the tortoises and thus the mineral composition of the tortoise blood [1,2,3,16]. A study in two different snake species [28] found a significant correlation between the Se concentration in the diet and the levels in the blood, indicating that dietary intake of this element plays an important role in blood levels. In the present study, there were significant differences in Cr, Cu, Fe, Mg, Mo, and Se levels between the four regions in which the tortoises were kept. A study in Hermann’s tortoises [16] found significant differences between the measured levels for Cu, Se, and Zn between tortoises kept in Germany, Turkey, and two different regions in France. However, since the tortoises studied here were kept under human care, it must also be considered that the animals were additionally fed with food plants from other regions, which can also have an effect on the plasma mineral concentrations [3]. It is therefore difficult to evaluate the cause of the regional differences noted.

The results show that all these factors can also interact with each other, as the season and its influence on the hormone balance also affect the sexes differently, especially in poikilothermic reptiles. On the other hand, different food plants grow at different times of the year, and these naturally also differ between the regions, so that these two factors also interact with each other.

## 5. Conclusions

This study measuring essential plasma trace elements in a large number of samples from captive Hermann’s tortoises in Central Europe using ICP-MS and established reference intervals for Cr, Co, Cu, Fe, Mg, Mn, Mo, Se, and Zn. The results of the study show that the sex of the animals as well as the season of blood collection and the region in which the tortoises are kept influence the amounts of trace elements in their blood and must therefore be taken into account when interpreting the results. For this reason, we calculated separate reference values for these factors in the present study. The results of this study serve as a basis for further studies, including on diseased animals, and help to identify and treat imbalances of the elements in the tortoise blood.

## Figures and Tables

**Table 1:** Calculated reference intervals for trace elements in heparinized plasma samples for all Hermann’s tortoises (*Testudo hermanni*) (*n* = 520) independent of sex, season, and region.

Element	Unit	Mean	SD	Minimum	Maximum	Median	10th Percentile	90th Percentile	Lower RI (CI)	Upper RI (CI)	D	*p*-Value
Chromium (Cr)	µg/L	0.49	0.49	0.05	7.19	0.45	0.05	0.87	0.1 (0.1–0.1)	1.3 (1.0–1.6)	NG	<0.001
Cobalt (Co)	µg/L	3.25	3.62	0.10	25.00	2.13	0.80	6.59	0.5 (0.4–0.6)	14.2 (13.2–15.7)	NG	<0.001
Copper (Cu)	mg/L	0.53	0.18	0.16	1.33	0.51	0.33	0.78	0.3 (0.2–0.3)	1.0 (0.9–1.0)	NG	<0.001
Iron (Fe)	mg/L	2.11	2.67	0.26	38.43	1.34	0.69	4.21	0.5 (0.5–0.6)	8.2 (7.1–10.2)	NG	<0.001
Magnesium (Mg)	mg/L	62.35	14.91	30.95	167.17	60.00	47.94	8.22	41.9 (39.6–43.1)	98.6 (90.1–106.1)	NG	<0.001
Manganese (Mn)	µg/L	9.17	8.52	0.50	54.30	6.40	2.70	20.40	1.7 (1.4–1.9)	34.9 (30.0–39.4)	NG	<0.001
Molybdenum (Mo)	µg/L	11.84	9.83	0.50	82.20	8.96	2.67	24.55	1.6 (1.4–1.8)	40.1 (31.1–45.1)	NG	< 0.001
Selenium (Se)	µg/L	15.67	8.97	0.70	79.30	13.80	6.25	27.10	4.2 (3.9–4.8)	38.00 (34.5–41.8)	NG	<0.001
Zinc (Zn)	mg/L	2.39	0.84	0.25	10.12	2.34	1.46	3.28	0.9 (0.5–1.1)	4.2 (3.8–4.3)	NG	<0.001

CI—90th confidence interval of the reference limits; D—distribution; G—Gaussian distribution; NG—non-Gaussian distribution; *p*-value of Anderson–Darling test for normal distribution, threshold *p* < 0.3.

**Table 2:** Calculated sex-specific reference intervals for trace elements in heparinized plasma samples for Hermann’s tortoises (*Testudo hermanni*) (*n* = 520) in which significant differences (*p* ≤ 0.05) were found between males (*n* = 318) and females (*n* = 202).

Element	Unit	Category	Mean	SD	Minimum	Maximum	Median	10th Percentile	90th Percentile	Lower RI (CI)	Upper RI (CI)	D	*p*-Value
Copper (Cu)	mg/L	Male	0.58	0.19	0.16	1.33	0.56	0.36	0.83	0.3 (0.3–0.3)	1.0 (0.9–1.2)	NG	<0.001
		Female	0.47	0.15	0.17	1.31	0.45	0.29	0.64	0.2 (0.2–0.3)	0.8 (0.7–1.0)	NG	<0.001
Magnesium (Mg)	mg/L	Male	58.81	11.90	30.95	158.34	56.59	46.89	71.36	41.3 (37.8–43.8)	85.9 (78.3–90.3)	NG	<0.001
		Female	67.93	17.31	34.78	167.17	65.08	50.80	87.35	41.9 (36.3–44.7)	112.9 (100.7–129.9)	NG	<0.001
Manganese (Mn)	µg/L	Male	6.38	5.23	0.50	54.30	5.10	2.50	11.20	1.6 (0.5–1.7)	16.4 (14.5–26.9)	NG	<0.001
		Female	13.56	10.61	1.00	54.10	10.00	3.30	29.10	2.1 (1.1–2.3)	43.7 (34.9–52.9)	NG	<0.001

CI—90th confidence interval of the reference limits; D—distribution; G—Gaussian distribution; NG—non-Gaussian distribution; *p*-value of Anderson–Darling test for normal distribution, threshold *p* < 0.3.

**Table 3:** Calculated season-specific reference intervals for trace elements in heparinized plasma samples for Hermann’s tortoises (*Testudo hermanni*) (*n* = 520) in which significant differences (*p* ≤ 0.05) were found between spring (*n* = 93), early summer (*n* = 210), and late summer (*n* = 217).

Element	Unit	Category	Mean	SD	Minimum	Maximum	Median	10th Percentile	90th Percentile	Lower RI (CI)	Upper RI (CI)	D	*p*-Value
Chromium (Cr)	µg/L	Spring	0.46	0.34	0.05	2.52	0.36	0.18	0.86	0.1 (0.1–0.1)	1.3 (1.0–2.5)	NG	<0.001
		Early summer	0.63	0.65	0.05	7.19	0.54	0.22	0.91	0.1 (0.1–0.1)	1.7 (1.3–5.7)	NG	<0.001
		Late summer	0.36	0.29	0.05	1.64	0.32	0.05	0.75	0.1 (0.1–0.1)	1.0 (0.9–1.4)	NG	<0.001
Copper (Cu)	mg/L	Spring	0.51	0.16	0.27	0.92	0.51	0.30	0.73	0.3 (0.3–0.3)	0.8 (0.8–0.9)	NG	0.043
		Early summer	0.56	0.17	0.16	1.31	0.53	0.37	0.78	0.3 (0.2–0.3)	1.0 (0.9–1.2)	NG	<0.001
		Late summer	0.52	0.20	0.17	1.33	0.48	0.31	0.79	0.2 (0.2–0.3)	1.0 (1.0–1.2)	NG	<0.001
Manganese (Mn)	µg/L	Spring	7.00	5.19	1.00	31.20	5.60	2.20	13.80	1.2 (1.0–1.7)	22.9 (15.7–31.2)	NG	<0.001
		Early Summer	11.14	9.25	0.50	52.90	7.50	3.20	24.85	1.5 (0.5–2.2)	36.5 (32.0–44.1)	NG	<0.001
		Late Summer	8.20	8.57	0.50	54.30	5.56	2.60	15.40	2.0 (1.7–2.2)	38.4 (26.9–54.1)	NG	<0.001
Molybdenum (Mo)	µg/L	Spring	8.08	4.68	1.55	20.90	7.78	2.48	16.0	1.8 (1.6–2.2)	17.4 (16.7–20.9)	NG	0.001
		Early Summer	10.03	9.47	0.50	82.20	7.75	1.85	19.85	1.2 (1.1–1.5)	29.3 (26.1–53.4)	NG	<0.001
		Late Summer	15.20	10.74	2.41	54.80	12.40	4.57	28.30	3.4 (2.7–3.8)	44.0 (40.0–51.1)	NG	<0.001
Selenium (Se)	µg/L	Spring	18.02	8.59	0.70	56.30	16.20	10.40	28.40	3.6 (0.7–5.7)	42.2 (33.3–56.3)	NG	<0.001
		Early Summer	17.72	10.54	3.60	79.30	16.20	6.03	30.45	4.5 (3.9–5.3)	43.3 (36.0–52.3)	NG	<0.001
		Late Summer	12.66	6.23	2.30	38.20	11.54	6.04	20.90	4.0 (2.5–5.0)	28.2 (25.6–33.3)	NG	<0.001

CI—90th confidence interval of the reference limits; D—distribution; G—Gaussian distribution; NG—non-Gaussian distribution; *p*-value of Anderson–Darling test for normal distribution, threshold *p* < 0.3.

**Table 4:** Calculated regional specific reference intervals for trace elements in heparinized plasma samples for Hermann’s tortoises (*Testudo hermanni*) in which significant differences (*p* ≤ 0.05) were found between each region of Germany and Lower Austria (north *n* = 58; west *n* = 42; east *n* = 149; south *n* = 271).

Element	Unit	Category	Mean	SD	Minimum	Maximum	Median	10th Percentile	90th Percentile	Lower RI (CI)	Upper RI (CI)	D	*p*-Value
Chromium (Cr)	µg/L	North	0.71	0.38	0.05	2.52	0.68	0.35	1.07	0.1 (0.1–0.3)	2.1 (1.3–2.5)	NG	<0.001
		West	0.25	0.13	0.05	0.71	0.23	0.11	0.38	0.1 (0.1–0.1)	0.7 (0.4–0.7)	NG	0.119
		East	0.54	0.30	0.05	1.87	0.51	0.25	0.91	0.1 (0.1–0.2)	1.4 (1.0–1.9)	NG	<0.001
		South	0.45	0.60	0.05	7.19	0.39	0.05	0.82	0.1 (0.1–0.1)	1.2 (1.0–1.7)	NG	<0.001
Copper (Cu)	mg/L	North	0.59	0.18	0.16	1.06	0.59	0.36	0.79	0.2 (0.2–0.3)	1.0 (0.9–1.1)	G	0.743
		West	0.50	0.16	0.22	0.94	0.48	0.32	0.71	0.2 (0.2–0.3)	0.9 (0.8–0.9)	G	0.457
		East	0.51	0.15	0.27	1.21	0.50	0.32	0.70	0.3 (0.3–0.3)	0.9 (0.8–1.2)	NG	0.027
		South	0.54	0.20	0.17	1.33	0.50	0.33	0.80	0.2 (0.2–0.3)	1.0 (1.0–1.2)	NG	<0.001
Iron (Fe)	mg/L	North	1.83	1.72	0.60	11.02	1.24	0.72	3.78	0.6 (0.6–0.7)	8.7 (4.9–11.0)	NG	<0.001
		West	3.41	2.56	0.64	15.70	2.95	1.45	4.86	0.6 (0.6–0.9)	15.1 (7.5–15.7)	NG	<0.001
		East	2.48	3.78	0.43	38.43	1.58	0.73	5.12	0.5 (0.4–0.7)	11.9 (7.1–38.4)	NG	<0.001
		South	1.77	1.94	0.26	17.46	1.12	0.64	3.46	0.5 (0.3–0.5)	7.6 (6.3–10.1)	NG	<0.001
Magnesium (Mg)	mg/L	North	56.53	9.65	30.95	75.62	56.42	44.28	69.14	34.2 (31.0–42.4)	75.2 (71.9–75.6)	G	0.907
		West	69.57	14.25	46.14	113.47	65.28	55.13	88.09	46.3 (46.1–53.1)	112.4 (90.5–113.5)	NG	0.014
		East	63.91	15.34	37.48	129.90	60.27	48.40	82.48	42.9 (37.5–46.2)	108.8 (90.1–129.9)	NG	<0.001
		South	61.63	15.18	34.78	167.17	59.43	47.76	75.21	40.3 (36.3–43.8)	97.4 (87.3–120.9)	NG	<0.001
Molybdenum (Mo)	µg/L	North	8.64	3.20	2.35	15.80	8.60	4.35	13.30	2.5 (2.4–3.4)	15.6 (13.7–15.8)	G	0.931
		West	14.47	14.84	1.21	82.20	9.18	2.68	27.60	1.2 (1.2–2.1)	80.0 (29.7–82.2)	NG	<0.001
		East	10.46	7.27	1.55	53.40	9.04	2.82	19.30	2.1 (1.6–2.4)	26.4 (21.5–53.4)	NG	<0.001
		South	12.87	10.75	0.50	54.80	8.96	2.31	27.10	1.4 (1.1–1.7)	42.3 (35.1–46.9)	NG	<0.001
Selenium (Se)	µg/L	North	13.19	8.38	3.60	43.30	10.65	5.80	23.70	3.7 (3.6–5.2)	39.5 (29.9–43.3)	NG	<0.001
		West	11.53	6.22	3.90	31.50	10.55	5.35	20.70	3.9 (3.9–4.8)	31.2 (21.4–31.5)	NG	0.001
		East	20.10	9.41	0.70	56.30	18.10	10.70	33.50	4.2 (0.7–5.3)	42.3 (38.0–56.3)	NG	<0.001
		South	14.40	8.32	2.30	79.30	12.64	6.35	24.25	4.3 (2.9–5.3)	33.5 (28.6–44.7)	NG	<0.001

CI—90th confidence interval of the reference limits; D—distribution; G—Gaussian distribution; NG—non-Gaussian distribution; *p*-value of Anderson–Darling test for normal distribution, threshold *p* < 0.3.

## Data Availability

The data that support the findings of this study are available from the corresponding author upon reasonable request.

## Supplementary Materials

The following supporting information can be downloaded at https://www.mdpi.com/article/10.3390/ani14152178/s1.
