# Supplementary material for: Trace Elements in Hermann’s Tortoises (Testudo hermanni) According to Sex, Season, and Sampling Region in Central Europe
Source: Animals (Basel). 2024 Jul 26;14(15):2178. doi: 10.3390/ani14152178 (PMC11311096; doi:10.3390/ani14152178)
Supplement: Supplementary file 1 [file animals-14-02178-s001.zip › animals-3101708-supplementary.pdf]

Supplementary material

Results of the Bonferroni post-hoc test for the factors saison and region for each element tested in the blood plasma of Hermann´s tortoises (*Testudo hermanni*) in central Europe.

Chromium

Saison	Saison	Saison	Difference in means (I-J)	Significance	95% Confidence interval
	Spring	Early summer	-0.174	0.005	-0.305 to -0.042
		Late summer	0.093	0.262	-0.038 to 0.224
	Early summer	Spring	0.174	0.005	0.042 to 0.305
		Late summer	0.267	<0.001	0.165 to 0.369
	Late summer	Spring	-0.093	0.262	-0.224 to 0.038
		Early summer	-0.267	<0.001	-0.369 to -0.165
Region	North	West	0.464	<0.001	0.228 to 0.700
		East	0.168	0.083	-0.012 to 0.348
		South	0.264	<0.001	0.096 to 0.433
	West	North	-0.464	<0.001	-0.700 to -0.228
		East	-0.296	<0.001	-0.499 to -0.092
		South	-0.200	0.038	-0.393 to -0.007
	East	North	-0.168	0.083	-0.348 to 0.012
		West	0.296	<0.001	0.092 to 0.499
		South	0.096	0.195	-0.023 to 0.215
	South	North	-0.264	<0.001	-0.433 to -0.096
		West	0.200	0.038	0.007 to 0.393
		East	-0.096	0.195	-0.215 to 0.023

Cobalt

Saison	Saison	Saison	Difference in means (I-J)	Significance	95% Confidence interval
	Spring	Early summer	-0.439	0.969	-1.504 to 0.627
		Late summer	-1.384	0.005	-2.445 to -0.324
	Early summer	Spring	0.439	0.969	-0.627 to 1.504
		Late summer	-0.945	0.019	-1.774 to -0.117
	Late summer	Spring	1.384	0.005	0.324 to 2.445
		Early summer	0.945	0.019	0.117 to 1.774
Region	North	West	-0.237	1.000	-2.148 to 1.674
		East	-0.558	1.000	-2.018 to 0.902
		South	-1.220	0.110	-2.584 to 0.145
	West	North	0.237	1.000	-1.674 to 2.148
		East	-0.321	1.000	-1.969 to 1.327
		South	-0.983	0.581	-2.547 to 0.582
	East	North	0.558	1.000	-0.902 to 2.018
		West	0.321	1.000	-1.327 to 1.969
		South	-0.662	0.414	-1.624 to 0.300
	South	North	1.220	0.110	-0.145 to 2.584
		West	0.983	0.581	-0.582 to 2.547
		East	0.662	0.414	-0.300 to 1.624

Copper

Saison	Saison	Saison	Difference in means (I-J)	Significance	95% Confidence interval
	Spring	Early summer	-0.051	0.053	-0.103 to 0.000
		Late summer	-0.017	1.000	-0.069 to 0.034
	Early summer	Spring	0.051	0.053	-0.000 to 0.103
		Late summer	0.034	0.126	-0.006 to 0.074
	Late summer	Spring	0.017	1.000	-0.034 to 0.069
		Early summer	-0.034	0.126	-0.074 to 0.006
Region	North	West	0.089	0.069	-0.004 to 0.182
		East	0.079	0.019	0.008 to 0.150
		South	0.055	0.173	-0.011 to 0.121
	West	North	-0.089	0.069	-0.182 to 0.004
		East	-0.010	1.000	-0.090 to 0.070
		South	-0.034	1.000	-0.110 to 0.042
	East	North	-0.079	0.019	-0.150 to -0.008
		West	0.010	1.000	-0.070 to 0.090
		South	-0.024	1.000	-0.071 to 0.022
	South	North	-0.055	0.173	-0.121 to 0.011
		West	0.034	1.000	-0.042 to 0.110
		East	0.024	1.000	-0.022 to 0.071

Iron

Saison	Saison	Saison	Difference in means (I-J)	Significance	95% Confidence interval
	Spring	Early summer	-0.193	1.000	-0.983 to 0.596
		Late summer	0.574	0.240	-0.212 to 1.359
	Early summer	Spring	0.193	1.000	-0.596 to 0.983
		Late summer	0.767	0.008	0.153 to 1.381
	Late summer	Spring	-0.574	0.240	-1.359 to 0.212
		Early summer	-0.767	0.008	-1.381 to -0.153
Region	North	West	-1.579	0.020	-2.995 to -0.163
		East	-0.651	0.669	-1.733 to 0.431
		South	0.065	1.000	-0.946 to 1.077
	West	North	1.579	0.020	0.163 to 2.995
		East	0.928	0.268	-0.293 to 2.149
		South	1.644	0.001	0.485 to 2.804
	East	North	0.651	0.669	-0.431 to 1.733
		West	-0.928	0.268	-2.149 to 0.293
		South	0.716	0.048	0.003 to 1.429
	South	North	-0.065	1.000	-1.077 to 0.946
		West	-1.644	0.001	-2.804 to -0.485
		East	-0.716	0.048	-1.429 to -0.003

Magnesium

Saison	Saison	Saison	Difference in means (I-J)	Significance	95% Confidence interval
	Spring	Early summer	-2.767	0.310	-6.838 to 1.304
		Late summer	-2.601	0.371	-6.652 to 1.450
	Early summer	Spring	2.767	0.310	-1.304 to 6.838
		Late summer	0.166	1.000	-2.998 to 3.330
	Late summer	Spring	2.601	0.371	-1.450 to 6.652
		Early summer	-0.166	1.000	-3.330 to 2.998
Region	North	West	-13.045	<0.001	-20.347 to -5.742
		East	-7.382	0.003	-12.960 to -1.804
		South	-5.096	0.059	-10.311 to 0.118
	West	North	13.045	<0.001	5.742 to 20.347
		East	5.663	0.106	-0.634 to 11.959
		South	7.948	0.003	1.972 to 13.925
	East	North	7.382	0.003	1.804 to 12.960
		West	-5.663	0.106	-11.959 to 0.634
		South	2.286	0.601	-1.390 to 5.961
	South	North	5.096	0.059	-0.118 to 10.311
		West	-7.948	0.003	-13.925 to -1.972
		East	-2.286	0.601	-5.961 to 1.390

Manganese

Saison	Saison	Saison	Difference in means (I-J)	Significance	95% Confidence interval
	Spring	Early summer	-4.150	<0.001	-6.368 to -1.932
		Late summer	-1.207	0.569	-3.415 to 1.000
	Early summer	Spring	4.150	<0.001	1.932 to 6.368
		Late summer	2.943	<0.001	1.219 to 4.667
	Late summer	Spring	1.207	0.569	-1.000 to 3.415
		Early summer	-2.943	<0.001	-4.667 to -1.219
Region	North	West	0.281	1.000	-3.698 to 4.261
		East	4.138	0.002	1.098 to 7.177
		South	3.665	0.004	0.823 to 6.506
	West	North	-0.281	1.000	-4.261 to 3.698
		East	3.856	0.018	0.425 to 7.287
		South	3.383	0.037	0.127 to 6.640
	East	North	-4.138	0.002	-7.177 to -1.098
		West	-3.856	0.018	-7.287 to -0.425
		South	-0.473	1.000	-2.476 to 1.530
	South	North	-3.665	0.004	-6.506 to -0.823
		West	-3.383	0.037	-6.640 to -0.127
		East	0.473	1.000	-1.530 to 2.476

Molybdenum

Saison	Saison	Saison	Difference in means (I-J)	Significance	95% Confidence interval
	Spring	Early summer	-1.949	0.248	-4.641 to 0.743
		Late summer	-7.124	<0.001	-9.802 to -4.445
	Early summer	Spring	1.949	0.248	-0.743 to 4.641
		Late summer	-5.175	<0.001	-7.267 to -3.083
	Late summer	Spring	7.124	<0.001	4.445 to 9.802
		Early summer	5.175	<0.001	3.083 to 7.267
Region	North	West	-5.831	0.009	-10.659 to -1.003
		East	-1.820	1.000	-5.508 to 1.868
		South	-4.233	0.007	-7.681 to -0.785
	West	North	5.831	0.009	1.003 to 10.659
		East	4.012	0.066	-0.152 to 8.175
		South	1.598	1.000	-2.353 to 5.550
	East	North	1.820	1.000	-1.868 to 5.508
		West	-4.012	0.066	-8.175 to 0.152
		South	-2.413	0.053	-4.844 to 0.017
	South	North	4.233	0.007	0.785 to 7.681
		West	-1.598	1.000	-5.550 to 2.353
		East	2.413	0.053	-0.017 to 4.844

Selenium

Saison	Saison	Saison	Difference in means (I-J)	Significance	95% Confidence interval
	Spring	Early summer	0.297	1.000	-1.916 to 2.510
		Late summer	5.356	<0.001	3.152 to 7.560
	Early summer	Spring	-0.297	1.000	-2.510 to 1.916
		Late summer	5.059	<0.001	3.337 to 6.781
	Late summer	Spring	-5.356	<0.001	-7.560 to -3.152
		Early summer	-5.059	<0.001	-6.781 to -3.337
Region	North	West	1.666	1.000	-2.304 to 5.636
		East	-6.905	<0.001	-9.938 to -3.872
		South	-1.208	1.000	-4.043 to 1.628
	West	North	-1.666	1.000	-5.636 to 2.304
		East	-8.571	<0.001	-11.994 to -5.147
		South	-2.873	0.118	-6.124 to 0.377
	East	North	6.905	<0.001	3.872 to 9.938
		West	8.571	<0.001	5.147 to 11.994
		South	5.697	<0.001	3.698 to 7.697
	South	North	1.208	1.000	-1.628 to 4.043
		West	2.873	0.118	-0.377 to 6.124
		East	-5.697	<0.001	-7.697 to -3.698

Zinc

Saison	Saison	Saison	Difference in means (I-J)	Significance	95% Confidence interval
	Spring	Early summer	-0.647	<0.001	-0.880 to -0.415
		Late summer	-0.284	0.010	-0.515 to -0.053
	Early summer	Spring	0.647	<0.001	0.415 to 0.880
		Late summer	0.363	<0.001	0.182 to 0.543
	Late summer	Spring	0.284	0.010	0.053 to 0.515
		Early summer	-0.363	<0.001	-0.543 to -0.182
Region	North	West	-0.075	1.000	-0.492 to 0.341
		East	0.266	0.164	-0.052 to 0.584
		South	0.356	0.010	0.058 to 0.653
	West	North	0.075	1.000	-0.341 to 0.492
		East	0.341	0.073	-0.018 to 0.701
		South	0.431	0.005	0.090 to 0.772
	East	North	-0.266	0.164	-0.584 to 0.052
		West	-0.341	0.073	-0.701 to 0.018
		South	0.090	1.000	-0.120 to 0.299
	South	North	-0.356	0.010	-0.653 to -0.058
		West	-0.431	0.005	-0.772 to -0.090
		East	-0.090	1.000	-0.299 to 0.120
